# Dancing Improves Emotional Regulation in Women With Methamphetamine Use Disorder But Use of a Cycle Ergometer Does Not

**DOI:** 10.3389/fnins.2021.629061

**Published:** 2021-06-29

**Authors:** Qian Tao, Chenping Zhang, Xiawen Li

**Affiliations:** ^1^Affiliated Sport School, Shanghai University of Sport, Shanghai, China; ^2^School of Psychology, Shanghai University of Sport, Shanghai, China; ^3^Department of Physical Education, Shanghai University of Medicine and Health Sciences, Shanghai, China

**Keywords:** dorsolateral prefrontal cortex, HbO2 concentration, negative images, emotional regulation, dancing

## Abstract

**Background:**

Emotional regulation is crucial to people who receive a diagnosis of methamphetamine (MA) use disorder. Although evidence that exercise improves emotional regulation is robust, little is known about whether exercise will improve emotional processing in women with MA use disorder.

**Methods:**

In the present study, 36 women with MA use disorder aged 20 to 34 years and residing in the Drug Rehabilitation Bureau of Mogan Mountain in Zhejiang province were assigned to 1 of 2 exercise intervention groups-dancing or stationary cycling. Both types of exercise were performed at 65–75% of the maximum heart rate for 30 min. Immediately before and after the exercise bout, the participants were asked to score their feelings using a nine-point Likert scale as they viewed emotionally negative, positive, or neutral images in blocks of 20 images each, for a total of 60 images. Concurrent with viewing the images and self-rating their emotions, the women also underwent functional near-infrared spectroscopy to assess changes in brain activity.

**Results:**

There were no significant differences in the demographic or MA use characteristics assessed for the women between the two exercise groups. We found main effect of image valence (*F*_2,33_ = 69.61, *p* < 0.01), significant interaction effect of time and image valence was found (*F*_2,33_ = 4.27, *p* < 0.05) and trend increase in the self-rated emotional scale score for viewing negative images in both groups after 30-min exercise intervention, and the dancing group presented more significant trends than cycling group. In addition, activation in the dorsolateral prefrontal cortex of dancers, but not of cyclists, while viewing negative images was significantly lower after vs. before dancing (*F*_2,33_ = 5.43, *p* < 0.05). This result suggested that 30 min of dancing decreased neural activity in women with MA use disorder while they viewed negative images specifically in a brain region known to guide the selection of appropriate behaviors, and to shift attention.

Taken together, the findings of this study suggest that for women with MA abuse disorder, 30 min of dancing, rather than of stationary cycling, may ameliorate negative emotional reactions by decreasing attention to negative stimuli.

## Introduction

Compared to amphetamine, methamphetamine (MA) has more adverse effects on both the central nervous system and the sympathetic nervous system (Davidson et al., 2001). MA targets the dopamine transporter and can cause emotional disorders. The association between emotional disorders and MA use appears to be bidirectional. Numerous study results have suggested that people who have emotional issues have higher risk for the use of drugs, including MA. For instance, one study found that the lifetime risk of drug abuse or dependence was 6.19 times more likely for individuals with vs. without emotional issues (Grant, 1995). In addition, other studies have found that MA users have a higher proportion of emotional disorders than non-users. Semple et al. (2005) reported that approximately 40% of their participants who were MA users also had moderate to severe depression, and greater intensity of MA use was correlated with higher levels of depressive symptoms in their study, even after controlling for demographic factors. Moreover, MA users are more likely to report a need for psychiatric help (Kalechstein et al., 2000). A negative emotional state may greatly hinder drug users’ intention to withdraw from drug use and may induce drug craving (Childress et al., 1994) and drug-seeking behaviors (Uslaner et al., 1999; Baker et al., 2004), which may lead to drug-taking relapse. Thus, improving the emotional state of MA users will help prevent drug relapse.

Exercise has been shown to effectively improve an individual’s emotional state. It has been suggested that exercise may promote emotional regulation by improving emotional self-efficacy and strengthening top-down executive control (Bernstein and Mcnally, 2017). Another study has shown that exercise improves the subjective evaluation of emotional stress and helps individuals recover from negative emotions (Bartholomew et al., 2005). Neuroscience research has shown that exercise activates brain areas involved in emotional regulation, for example, the ventral and dorsal prefrontal cortex (Etkin et al., 2015; Edwards et al., 2017). Among all the exercise, cycling and dancing were suggested having reduced the attention toward drugs and increased MA-users’ vital capacity (Lv et al., 2019; Xu et al., 2019). However, whether cycling and dancing can improve emotional regulation in MA users and their different effect is still unclear.

Among the brain areas involved in emotional processing, the prefrontal cortical region has been shown by numerous studies to participate in different emotional processes. For example, the orbitofrontal cortex (OFC) is thought to integrate affective valuation of specific stimuli and inputs from other regions associated with emotional and social processing, such as the amygdala and medial temporal lobe systems (Ochsner et al., 2002; Ongur et al., 2003; Cunningham et al., 2011) to then evaluate stimuli in a context and in a goal-dependent manner (Oya et al., 2005; Roy et al., 2012). Ochsner et al. (2004) reported that the ventrolateral prefrontal cortex and the dorsolateral prefrontal cortex (dlPFC) are associated with responses to negative affective stimuli and that they modulate the amygdala. The frontopolar area plays an important role in complicated cognitive processes, one of which is social cognition (Burgess et al., 2007).

Based on the observations of these previous studies, the aim of the present work was to explore whether acute exercise can improve emotional processing in MA users and to investigate the mechanisms underlying any exercise-induced alteration in emotional regulation. To that end, we assessed the self-reported emotional state as well as neural activity in prefrontal lobe structures, including the OFC, ventrolateral prefrontal cortex, dlPFC, and frontopolar area, by using functional near-infrared spectroscopy (fNIRS) of women with MA use disorder as they viewed positive, neutral, and negative emotional stimuli (images). The self-reported emotional state and neural activity assessments were conducted before and after participants performed 1 of 2 types of exercise for 30 min: dancing or using a cycle ergometer (a stationary bicycle).

## Materials and Methods

### Participants

In total, 39 women who had MA use disorder were recruited from the Drug Rehabilitation Bureau of Mogan Mountain in Zhejiang province (No. 20190811). All participants had received a diagnosis of MA use disorder as described using the Diagnostic and Statistical Manual of Mental Disorders (fifth edition) criteria and were not dependent on other substances (such as marijuana, heroin, and cocaine). Participants also met the following criteria: (1) aged 18–35 years; (2) abstinent and receiving treatment for at least 3 months; (3) no history of mental illness, brain trauma, alcohol dependence, or other diseases that affect the structure or function of the brain, and no recent taking of any psychiatric drugs; (4) educational level of elementary school or above; and (5) less than two substance abuse withdrawal experiences. The study was performed in accordance with the Declaration of Helsinki and was approved by the Shanghai University of Sport Ethics Committee (No. 2015007). All participants provided written informed consent prior to the study.

### Procedure

**FIGURE 1:**
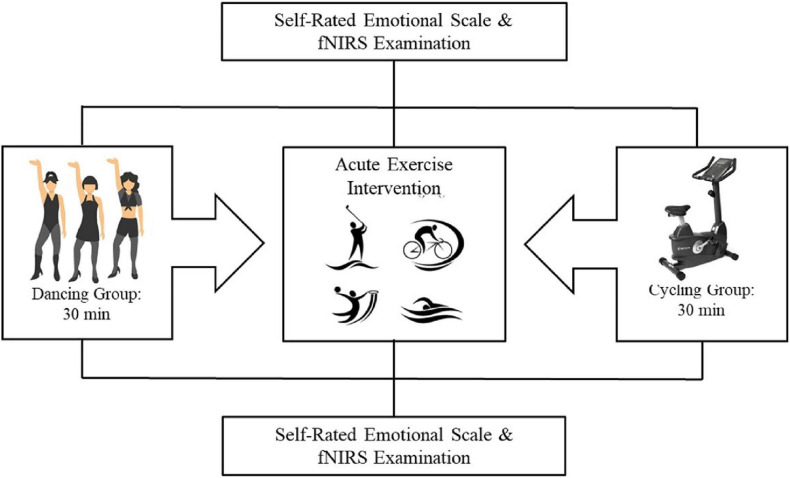
Overview of the experimental paradigm.

The participants were assigned to either the dancing group or the stationary cycling group such that their demographic and MA usage characteristics were balanced. The procedure consisted of participants viewing images on a computer screen and providing their emotional responses to those images on a self-rated emotional scale while undergoing fNIRS, both immediately before and after the 30-min exercise intervention (dancing or stationary cycling). The entire procedure took approximately 40 min for each participant to complete (Figure 1).

### Stimuli and Self-Rated Emotional Assessment

We selected 75 images from the Chinese Affective Picture System (Bai et al., 2005). Of the 75 images, 25 were considered emotionally positive, 25 emotionally neutral, and 25 emotionally negative. Prior to the start of the formal experiment, participants viewed 15 images to practice using the self-rating scale. In the formal experiment, the remaining 60 images (consisting of 20 positive, 20 neutral and 20 negative images) were viewed. The same 60 images were viewed before and after the exercise intervention. Each image was assessed for the two main dimensions of emotion, that is, its valence and arousal, by participants using a nine-point Likert self-assessment scale: a score of one indicated that the participant felt very unpleasant; five, the participant felt neutral; and nine, the participant felt very pleasant. Image valence refers to the emotional valence of images, including positive emotions, negative emotions and neutral emotions; and image arousal means how degree the emotion is aroused by the image.

**FIGURE 2:**
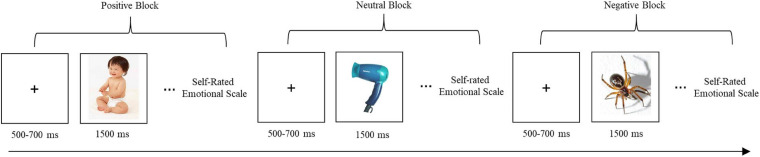
Image presentation and self-rating procedure. Blocks were presented in random order among the participants.

Image presentation was conducted using a block design that included three blocks: one block contained 20 positive images; one block contained 20 neutral images; and one block contained 20 negative images (Figure 2). Each image was presented for 1,500 ms, with a 500–700 ms interval between image presentations in which participants viewed a plus sign displayed on the screen. Blocks were presented in random order among participants. Participants were asked to rate their feelings after viewing each image. They were given a brief rest before the next block of images was presented.

### Acute Exercise Intervention

Two different types of acute exercise intervention were used. Participants in the dancing group were instructed to dance under the supervise of professional teacher for 30 min, and those in the stationary cycling (SH-5000U; Shu hua, Quanzhou, China) group were asked to cycle for the same amount of time. Both exercise interventions included a 5-min warm-up before the exercise and 5 min of stretching after the exercise. Individuals in both groups were asked to control their heart rate between 65–75% of their maximum heart rate based on their age. The maximum heart rate was calculated using the Tanaka formula: 208 – (0.7 × age) (Tanaka et al., 2001). A Suunto Smart Sensor heart rate monitor was used to record each participant’s heart rate.

### fNIRS Data Acquisition

A multichannel, continuous wave fNIRS instrument (NIRScout, NIRx Medical Technologies LLC, Minneapolis, MN, United States) was used to monitor brain hemodynamic activity, specifically, the changes in brain tissue concentrations of oxyhemoglobin (HbO2) associated with neuronal activity and tissue perfusion, as participants viewed the selected images and self-rated their emotional responses to those images. Dual wavelength (760 nm and 850 nm) continuous-wave near-infrared diffuse tomographic measurements were obtained with an acquisition sampling rate of 7.81 Hz. We placed 8 sources and seven optical probe detectors on the scalp to assess PFC and the OFC activity using a 20-channel configuration (for details, see Wang et al., 2019). The probes were positioned according to the International 10–20 electroencephalogram system, with some adjustment to ensure that each emitter was 3 cm from its corresponding detector. The fNIRS channel locations were positioned to capture the relevant brain activity based on previous studies (Tsuzuki et al., 2007; Okamoto et al., 2009).

### Hemodynamic Imaging Analyses

After acquiring the fNIRS data, we analyzed only the change in the HbO2 concentration (Δ[HbO2]) signal, rather than the deoxygenated hemoglobin signal, because the former gives a better signal-to-noise ratio than the deoxygenated hemoglobin signal (Niu et al., 2013; Schaeffer et al., 2014). The Homer2 software (MGH-Martinos Center for Biomedical Imaging; Boston, MA, United States) (Huppert et al., 2009) script for MATLAB (MathWorks; Natick, MA, United States) was used to analyze [HbO2]. The fNIRS data were first converted into optical density data and then into ΔHbO2 using the modified Beer-Lambert law (Cope et al., 1988). Motion artifacts were then removed using NIRlab software. A bandpass filter between 0.01 and 0.1 HZ was applied to remove any baseline drift and physiological noise (e.g., heartbeat) after removing discontinuous shifts from the time-series data. The mean [HbO2] was calculated for the dancing group and separately for the stationary cycling group for further analysis.

### Statistical Analysis

We used SPSS, version 22.0 (SPSS Inc.; Chicago, Il, United States) to assess the behavioral data. Independent-samples *t*-tests were used to compare the differences in characteristics between the dancing and cycling groups (e.g., age, educational level, and self-rated emotional feelings). We applied two-way repeated-measures analyses of variance to determine the main effects and interactions among the effects for the two exercise interventions, self-reported emotional dimension scores, and brain activation as assessed through hemodynamic changes (i.e., HbO2). All data are reported as means ± standard deviations (SDs). A two-sided *p* < 0.05 was considered statistically significant.

## Results

### Demographic and MA Use Characteristics of Participants and Exercise Intensity

All 36 female participants were included in the final analyses. Their ages ranged from 20 to 34 years (26.85 ± 4.20 years). We assigned 17 women to the dancing group and the other 19 women to the cycling group. We balanced the two groups for age, educational level, MA use duration, MA use dosage, and MA use frequency. None of those variables were found to be significantly different between the two groups (Table 1). All of the participants had no prior experience in either of the exercise, and they were assigned to each group based on their demographic and MA use characteristics, not on their preference.

**TABLE 1:** Demographic and methamphetamine use characteristics of all participants, by group (*N* = 36).

Characteristic	Dancing group (*n* = 17)	Cycle ergometer group (*n* = 19)	*p*-Value
Age, year (mean ± SD)	26.80 ± 4.15	26.89 ± 4.37	0.99
Education level, year (mean ± SD)	7.90 ± 1.65	8.37 ± 1.80	0.45
MA Use			
Duration, year (mean ± SD)	6.50 ± 3.80	6.42 ± 2.99	0.43
Dosage, g/dose (mean ± SD)	0.71 ± 0.55	0.57 ± 0.43	0.49
Frequency, days/week (mean ± SD)	4.61 ± 2.55	5.34 ± 2.35	0.26

The heart rate of each group was controlled between 65–75% of Maximum heart rate, it was 69.95 and 69.69%, respectively, in dancing group and cycling group during the exercise. There was no difference of the heart rate at baseline of both groups (72.95 ± 8.07 bpm) for dancing group and 75.11 ± 6.46 bpm for cycling group, *p* = 0.38), or during the exercise (132.38 ± 3.83 bpm for dancing group and 131.83 ± 4.04 bpm for cycling group, *p* = 0.67).

### Emotional Response

Before the exercise intervention, the participants used a nine-point Likert scale to self-rate their emotional responses to 60 images, 20 of which were considered positive, 20 negative, and 20 neutral. There was a significant difference in the emotional dimension of valence among the three types of images: positive vs. negative, positive vs. neutral, and negative vs. neutral (all *p* < 0.001). Although no difference in the emotional dimension of arousal was found between images considered positive and negative (*p* = 0.28), both positive and negative images were rated by participants as being significantly higher in arousal than neutral images (*p* < 0.001 for both comparisons) (Table 2).

**TABLE 2:** Valence and arousal scores of images considered positive, negative, or neutral before exercise intervention in 36 women with methamphetamine use disorder.

Emotional type	Positive	Neural	Negative
Valence^*a*^ (mean ± SD)	6.84 ± 0.40	4.99 ± 0.18	2.49 ± 0.84
Arousal^*b*^ (mean ± SD)	6.80 ± 1.33	2.49 ± 0.84	6.21 ± 0.53

*ANOVA was conducted to compare the differences among the affective images.*

*^*a*^Valence comparison: positive vs. negative, positive vs. neutral, and negative vs. neutral; all *p* < 0.001.*

*^*b*^No difference in arousal was found between images considered positive and negative (*p* = 0.28); both positive and negative images were rated by participants as being significantly higher in arousal than neutral images (*p* < 0.001) for both comparisons.*

Two-way repeated-measures was employed to explore the main effect of time (before vs. after exercise intervention), image valence (positive, neural, or negative) and their interactions on Self-Rated Emotional Assessment Scale Score. The main effect of image valence was significant (*F*_2,33_ = 69.61, *p* < 0.01), specifically, participants had highest Self-Rated Emotional Assessment Scale Score after watching positive images, then were the neutral images, and had the lowest score after watching negative images. There was no main effect of time (*F*_2,33_ = 0.97, *p* = 0.76) on Self-Rated Emotional Assessment Scale Score.

**FIGURE 3:**
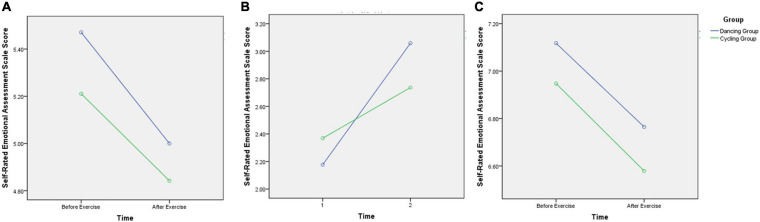
Comparison of self-rated emotional scale score for viewing different valence images before and after exercise intervention in the two groups. **(A)** The self-rated emotional scale score for viewing neural images before and after exercise intervention in the two groups; **(B)** The self-rated emotional scale score for viewing negative images before and after exercise intervention in the two groups; **(C)** The self-rated emotional scale score for viewing positive images before and after exercise intervention in the two groups. Though there was no interaction effect of time and group (*F*_2,33_ = 0.19; *p* = 0.67), we found a trend increase in the self-rated emotional scale score for viewing negative images in both groups after 30-min exercise intervention, and the dancing group presented more significant trends than cycling group.

For the Self-Rated Emotional Assessment Scale Score, a significant interaction effect of time and image valence was found (*F*_2,33_ = 4.27, *p* < 0.05), the participants had significant higher score after the exercise intervention when watching negative images. No interaction effect of image valence and group (*F*_2,33_ = 0.02; *p* = 0.98). Though there was no interaction effect of time and group (*F*_2,33_ = 0.19; *p* = 0.67), we found a trend increase in the self-rated emotional scale score for viewing negative images in both groups after 30-min exercise intervention, and the dancing group presented more significant trends than cycling group. By contrast, there was no such trends of both groups on the self-rated emotional scale scores for viewing positive or neutral images before and after the acute exercise intervention (Figure 3). In addition, no interaction was found among the main effects of image valence, group, or time (before vs. after exercise intervention) (*F*_2,3__3_ = 0.36; *p* = 0.70).

### Hemodynamic Response

**FIGURE 4:**
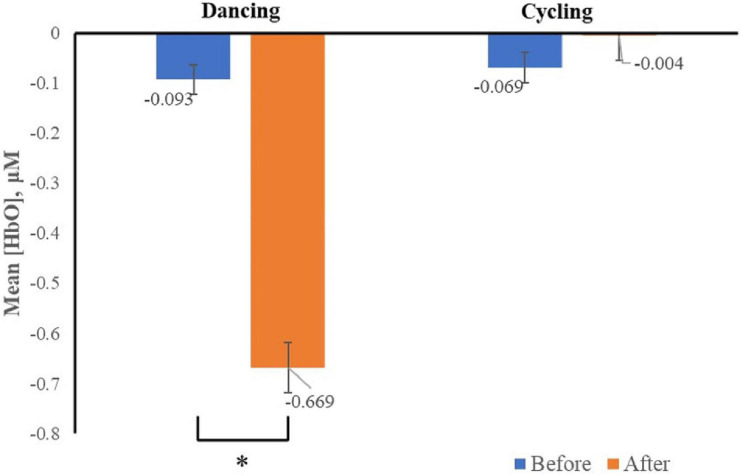
Mean changes in oxyhemoglobin concentration (HbO), which represents neural activation, for channel two as participants viewed negative stimuli before and after acute exercise, by group. Channel two was positioned to capture activity in the dlPFC, bars represent participant means, and vertical lines represent standard errors of the mean. *p < 0.05.

The Δ[HbO2], which represents neural activation was recorded. We only found the interaction effect of time and group in channel two (*F*_2,33_ = 5.43, *p* < 0.05), specifically, the Δ[HbO2] was significantly decreased in channel two after dancing (vs. before dancing) when dancers viewed images associated with negative emotion (Figure 4).

No main effect of time (*F*_2,33_ = 3.65, *p* = 0.07), image valence (*F*_2,33_ = 0.043 *p* = 0.96) was found in all channels. There was no interaction between image valence and group (*F*_2,33_ = 0.24, *p* = 0.79), or between image valence and time (*F*_2,33_ = 0.28, *p* = 0.75) in all channels. In addition, no interaction was found among the main effects of image valence, group, or time (before vs. after exercise intervention) (*F*_2,33_ = 1.61; *p* = 0.22) in all channels. This result suggested that 30 min of dancing, rather than cycling significantly decreased the neural response to negative images in women with MA use disorder.

## Discussion

It has been suggested that negative emotional experiences have a greater impact than positive ones, both in the short- and long-term (Baumeister et al., 2001). This postulation has been supported by data on clinical disorders, specifically, that people who have problems regulating negative emotions have much higher risk of having mental disorders than those who have problems regulating positive emotions (American Psychiatric Association, 1995). Diagnostic and Statistical Manual of Mental Disorders. 4th ed. The main finding of our study was that acute exercise appeared to regulate emotion in women with MA use disorder by reducing the attention given to negative stimuli, both in terms of a behavioral response and brain activation. Our results indicated that 30 min of dancing had the trend in increasing participants’ self-rating of their feelings and decreased the neural activation associated with channel two when the participants viewed negative images. These results suggested that 30 min of dancing decreased an individual’s negative reaction when viewing negative images, and that this decrease may be attributable to decreased attention to negative stimuli.

Channel two was positioned to capture activity in the dlPFC, which has been suggested to be associated with attention shifting (Wager et al., 2004). Numerous studies have indicated that the dlPFC extracts important information (Wallis et al., 2001)-especially information that is behaviorally relevant (Rainer et al., 1998)-among mass data, indicating that the dlPFC is involved in information processing. Miller and Cohen (2001) hold that the dlPFC plays an important role in judging situational contexts and will guide the selection of appropriate behaviors. Thus, our finding of decreased activation in the dlPFC suggests that the women in our study were paying less attention to negative images. This decrease in attending to negative images would explain why participants had higher scores on a self-rated emotional scale after 30 min of dancing. Thus, dancing improved the feelings of women with MA use disorder by reducing their attention to negatives stimuli.

Our results also showed that not all acute exercise improved participants’ emotional states. Despite participants exercising for the same amount of time and maintaining the same heart rate level, dancing but not stationary cycling, impacted their emotions and brain activation. Previous studies have also found that different types of sports lead to different effects (Baches et al., 2018), that is, not all types of exercise improve people’s physical or mental health. For example, one study indicated that dance, tai chi, and running significantly improved olfactory function in people, whereas walking did not have the same effect (Zhang et al., 2020). The results of the difference in the two types of exercise examined in the present study may be because dancing requires more cognition than stationary cycling, and brain regions involved in cognition overlap with those involved in emotional regulation, including the PFC. Given that our study showed that not all types of exercise improve emotional regulation among women who have MA use disorder, the exercise type should be considered in future studies.

There are some limitations that should be taken into account when interpreting the results of the present study. First, there was no control group, we would suggest to have control group in future study, thus we could see the different effects of dancing and cycling on different participants cohorts. However, the aim of our study was to explore the effects of two types of acute exercise on emotional regulation in women with MA use disorder, and women were assigned to each group such that there were no differences in the baseline demographic or MA usage characteristics examined between the 2 groups. Second, we examined the effects of only an acute exercise intervention; future studies should consider longer-term exercise interventions on emotional regulation. Third, the two groups are not randomly assigned, we balanced the two groups for age, educational level, MA use duration, MA dosage, and MA use frequency to avoid the impact of these factors.

## Conclusion

Emotional regulation, especially the ability to regulate negative emotions, is critical to individuals who have MA use disorder to help prevent drug relapse. The present study found that 30 min of dance exercise, but not stationary cycling, increased self-rated emotions among women with MA use disorder by reducing their attention to negative stimuli.

## Data Availability Statement

The original contributions presented in the study are included in the article/supplementary material, further inquiries can be directed to the corresponding author/s.

## Ethics Statement

The studies involving human participants were reviewed and approved by ethics committee of the Shanghai University of Sport (No. 2015007). The patients/participants provided their written informed consent to participate in this study.

## Author Contributions

XL designed the experiment and final approved it. XL and QT conducted the experiment. CZ and QT analyzed the data and drafted the manuscript. All authors contributed to the article and approved the submitted version.

## Conflict of Interest

The authors declare that the research was conducted in the absence of any commercial or financial relationships that could be construed as a potential conflict of interest.
